# Arabidopsis Fatty Acid Desaturase FAD2 Is Required for Salt Tolerance during Seed Germination and Early Seedling Growth

**DOI:** 10.1371/journal.pone.0030355

**Published:** 2012-01-18

**Authors:** Jiantao Zhang, Hua Liu, Jian Sun, Bei Li, Qiang Zhu, Shaoliang Chen, Hongxia Zhang

**Affiliations:** 1 National Key Laboratory of Plant Molecular Genetics, Shanghai Institute of Plant Physiology and Ecology, Chinese Academy of Sciences, Shanghai, China; 2 College of Biological Sciences and Technology, Beijing Forestry University, Beijing, China; National Taiwan University, Taiwan

## Abstract

Fatty acid desaturases play important role in plant responses to abiotic stresses. However, their exact function in plant resistance to salt stress is unknown. In this work, we provide the evidence that FAD2, an endoplasmic reticulum localized *ω*-6 desaturase, is required for salt tolerance in Arabidopsis. Using vacuolar and plasma membrane vesicles prepared from the leaves of wild-type (Col-0) and the loss-of-function Arabidopsis mutant, *fad2*, which lacks the functional FAD2, we examined the fatty acid composition and Na^+^-dependent H^+^ movements of the isolated vesicles. We observed that, when compared to Col-0, the level of vacuolar and plasma membrane polyunsaturation was lower, and the Na^+^/H^+^ exchange activity was reduced in vacuolar and plasma membrane vesicles isolated from *fad2* mutant. Consistent with the reduced Na^+^/H^+^ exchange activity, *fad2* accumulated more Na^+^ in the cytoplasm of root cells, and was more sensitive to salt stress during seed germination and early seedling growth, as indicated by CoroNa-Green staining, net Na^+^ efflux and salt tolerance analyses. Our results suggest that FAD2 mediated high-level vacuolar and plasma membrane fatty acid desaturation is essential for the proper function of membrane attached Na^+^/H^+^ exchangers, and thereby to maintain a low cytosolic Na^+^ concentration for salt tolerance during seed germination and early seedling growth in Arabidopsis.

## Introduction

High-level fatty acid (FA) unsaturation of the membrane is a common feature in plant cells [1]. Two types of fatty acid desaturases are responsible for fatty acid unsaturation. Fatty acid desaturase-2 (FAD2) of the endoplasmic reticulum (ER) and fatty acid desaturase-6 (FAD6) of the plastids encode two ω-6 desaturases that convert oleic acid (18∶1) to linoleic acid (18∶2) by inserting a double bond at the ω-6 position. Whereas fatty acid desaturase-3 (FAD3) of the ER and fatty acid desaturase-7 (FAD7) or fatty acid desaturase-8 (FAD8) of the plastids encode three ω-3 desaturases which convert linoleic acid (18∶2) to linolenic acid (18∶3) by inserting a double bond at the *ω*-3 position.

Recent studies have shown that increased production of trienoic fatty acids is a response connected with cold acclimation. In *Arabidopsis thaliana*, expression of *FAD8* was strongly inducible by low temperature [2]. In tomato, expression of *LeFAD7*, was inducible by chilling stress (4°C), but inhibited by high temperature (45°C) in leaves [3]. In tobacco, fatty acid desaturation during chilling acclimation is one of the factors involved in conferring low-temperature tolerance to young leaves [4]. In transplastomic tobacco, expression of a fatty acid desaturase gene from either wild potato *Solanum commersonll* or the cyanobacterium *Anacystis nidulans* altered the fatty acid profiles, and improved the cold tolerance [5]. Transgenic tobacco plants overexpressing *FAD7* also showed enhanced cold tolerance [6], [7], whereas those with silenced *FAD7* gene contained a lower level of trienoic fatty acids than wild-type plants, and were more tolerant to high temperature [8]. Furthermore, antisense expression of the Arabidopsis *FAD7* reduced salt/drought tolerance in transgenic tobacco plants [9], whereas overexpression of either *FAD3* or *FAD8* increased tolerance to drought in tobacco plants, and to osmotic stress in cultured cells [10]. Similar growth phenotypes were also observed in transgenic tomato plants expressing the tomato antisense *LeFAD7* gene [3].

FAD2 is the main enzyme responsible for polyunsaturated lipid synthesis in developing seeds of oil crops. The *fad2* mutants of Arabidopsis are deficient in activity of the endoplasmic reticulum oleate desaturase. When grown at low temperature, the seed development of *fad2* mutant was impaired [11]. However, *Saccharomyces cerevisiae* cells overexpressing the Arabidopsis *FAD2* showed greater resistance to 15% ethanol than did the control cells [12]. In cotton, the expression of *FAD2* is regulated by low temperature and light [13].

Polyunsaturated fatty acid contributes to maintenance of low temperature tolerance in plant [14]. The membrane lipids of Arabidopsis *fad6* mutant had elevated levels of monounsaturated fatty acids, and diminished levels of polyunsaturated fatty acids [15], and mutants *fad5*, *fad6* and *fad3*:*fad7*:*fad8* were more susceptible to photoinhibition than were the wild-type plants when subjected to chilling stress [14]. Previously, we reported that *FAD6* is an important component in plant response to salt stress [16]. Here, we show that the loss-of-function mutant of FAD2 (*fad2*) was hypersensitive to salt stress, and this hypersensitivity to salt is caused by the reduced vacuolar/plasma Na^+^/H^+^ antiporter activity and thereby an increased cytosolic Na^+^ accumulation in the mutant.

## Results

### *FAD2* is ubiquitously expressed in Arabidopsis

**Figure 1:**
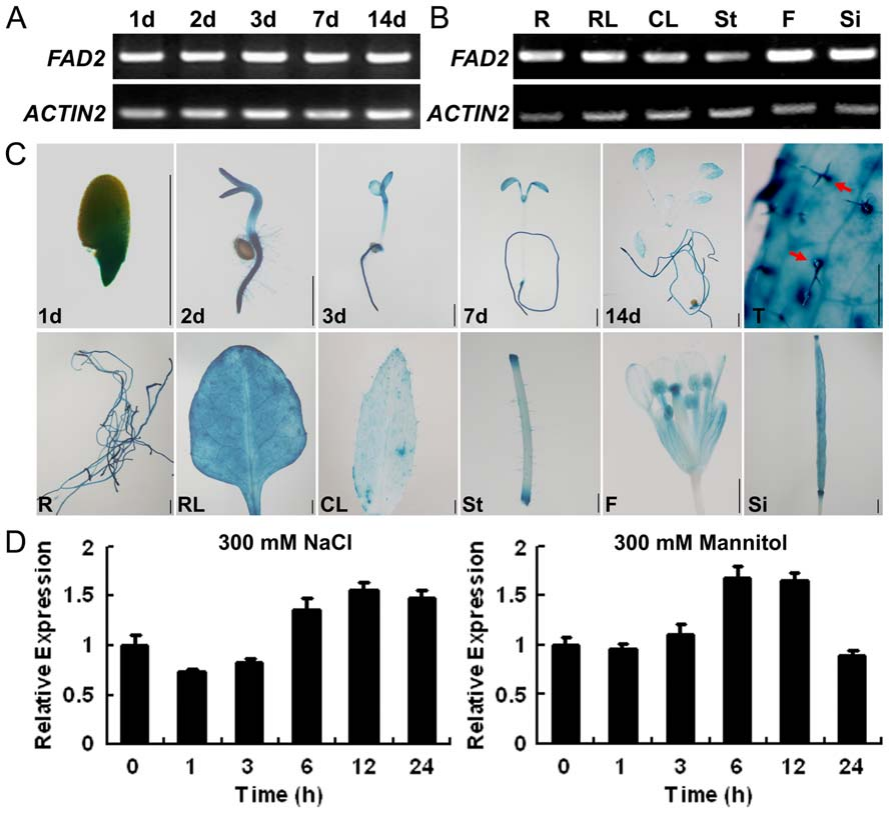
Expression analyses of *FAD2* in wild-type Col-0 plants. (A) and (B) RT-PCR analyses of *FAD2* transcripts. R, root; RL, rosette leaf; CL, cauline leaf; St, stem; F, flower; Si, silique. (C) GUS expression in ProFAD2:GUS transgenic plants. The upper panels show a 1-, 2-, 3-, 7-, 14-day-old seedling, and a rosette leaf (T) of five-week-old plants, respectively. The lower panels depict root (R), rosette leaf (RL), cauline leaf (CL), stem (St), flower (F) and silique (Si) of five-week-old plants, respectively. Red arrows indicate the high GUS expression in trichomes. Scale bar  =  1 mm. (D) Quantitative real-time PCR analyses of *FAD2* upon treatment with different stresses. Eight-day-old seedlings were treated with 300 mM NaCl or 300 mM mannitol. The unstressed expression level was assigned a value of 1. Data represents the average of three independent experiments ±SE.

As a first step to understand the possible biological functions of FAD2, we examined the expression patterns of *FAD2* gene in wild-type Arabidopsis grown under normal or different abiotic stress conditions by RT-PCR and quantitative real-time PCR. *FAD2* mRNA was ubiquitously present in seedlings (Fig. 1A) and various tissues, including roots, rosette leaves, cauline leaves, stems, flowers and siliques (Fig. 1B). We also generated *FAD2* promoter-GUS (ProFAD2:GUS) transgenic plants. The *FAD2* promoter fragment was used to drive the GUS expression in Arabidopsis. The expression pattern of ProFAD2:GUS in transgenic Arabidopsis plants was investigated. GUS activity was detected in seedlings and various tissues (Fig. 1C).

### Expression of *FAD2* is stress regulated

FAD2 expression under osmotic or both ionic/osmotic stress was examined using quantitative PCR (Fig. 1D). Upon treatment with 300 mM NaCl, the transcript level of *FAD2* increased after 6 h and reached a maximum level at 12 h. Upon treatment with 300 mM mannitol, the transcript level of *FAD2* increased at 3 h, reached maximum at 6 h, and declined at the 24 h time point (Fig. 1D). The induced *FAD2* transcript was transient as shown for the induction of many other signaling components, such as CBL1, CIPK3, and CIPK9 [17]–[19].

### Heterologous expression of FAD2 enhanced salt tolerance in yeast

**Figure 2:**
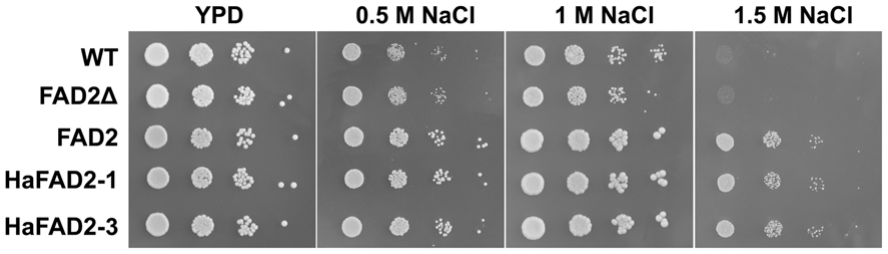
Heterologous expression of Arbidopsis *FAD2* in *S. cerevisiae*. Cells of the wild-type yeast strain W303-1 were transformed with *pVTFAD2Δ* (*FAD2Δ*), *pVTFAD2* (*FAD2*), *pVTHaFAD2-1* (*HaFAD2-1*) and *pVTHaFAD2-3* (*HaFAD2-3*), respectively. The mutated *FAD2Δ* gene was used as negative controls, whereas the sunflower *HaFAD2-1* and *HaFAD2-3* were used as positive controls. Photos were taken at 2 (YPD and 0.5 M) or 5 (1 M and 1.5 M) days. Photos are representatives of three independent experiments. WT, wild-type W303-1; FAD2Δ, W303-1 harboring the mutated *FAD2Δ* gene; FAD2, W303-1 harboring the functional *FAD2* gene; HaFAD2-1 and HaFAD2-3, W303-1 harboring the functional sun flower *FAD2* gene.

Unlike other eukaryotic organisms that can synthesize dienoic fatty acids, *Saccharomyces cerevisiae* can introduce only one double bond at the Δ9 position. Heterologous expression of the Arabidopsis FAD2 or two sunflower (*Helianthus annuus*) oleate Δ12 desaturases (HaFAD2-1 and HaFAD2-3) increased the content of dienoic fatty acids, especially 18∶2, as well as the fluidity of the yeast membrane [20], [21]. Although the total fatty acid content remained constant, the level of monounsaturated fatty acids decreased [20], [21]. We expressed *FAD2* in yeast cells of the wild-type W303-1 strain and analyzed its effect on salt tolerance. HaFAD2-1 and HaFAD2-3 were used as positive controls. Compared to those of wild-type W303-1 and FAD2Δ, growth inhibition of yeast cells expressing FAD2, HaFAD2-1 or HaFAD2-3 was much less under different concentrations of NaCl treatment at 30°C (Fig. 2). Thus, expression of FAD2 increased NaCl tolerance in yeast.

### Increased monounsaturated and diminished polyunsaturated fatty acid levels in *fad2*

**Figure 3:**
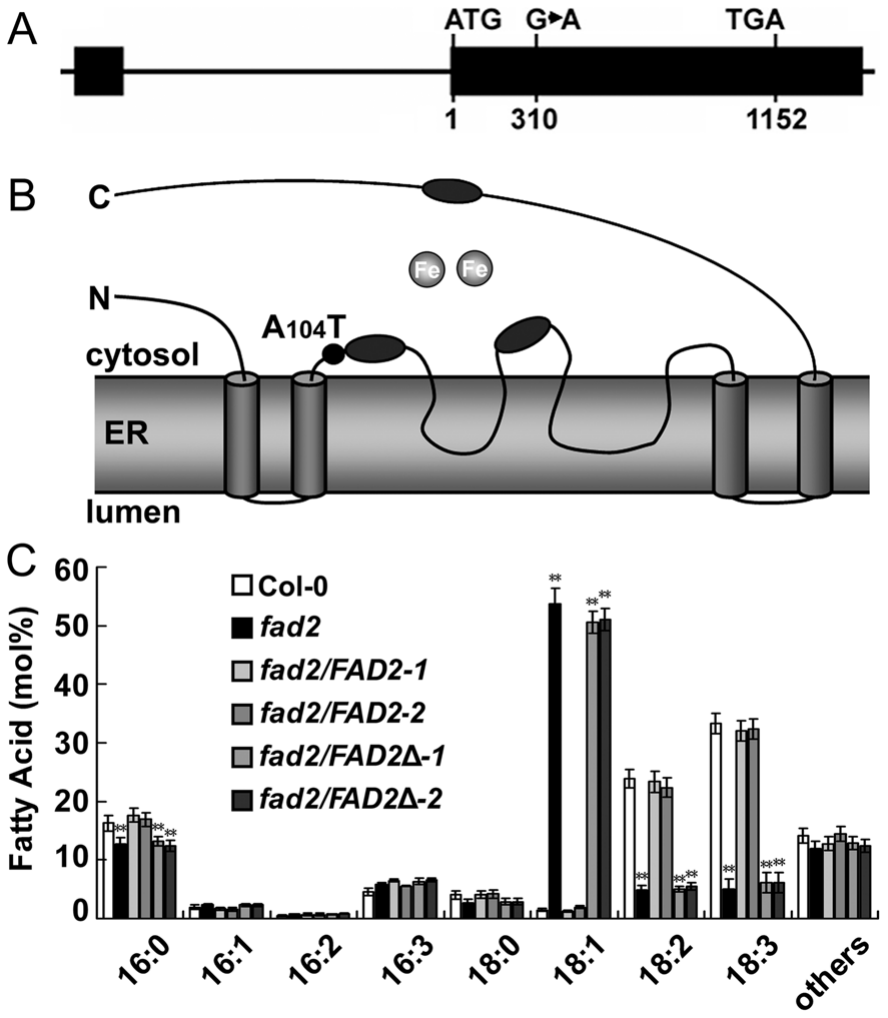
*FAD2* gene mutation and fatty acid analyses in *fad2*. (A) Intron-exon organization of the Arabidopsis *FAD2* gene (not to scale). The mutation site was indicated. Solid boxes and lines represent exons and introns, respectively. (B) The amino acid mutation site in the predicted topology of FAD2 protein. Three His boxes (black ellipses) oriented on the cytosolic side of the membrane coordinate two iron atoms (gray circles) at the active site of the protein. The mutation site was indicated (from A_104_ to T_104_). (C) Fatty acid analyses of Col-0, *fad2* and *fad2* mutant complemented with the functional wild-type *FAD2* or the mutated *FAD2Δ* gene. Col-0, wild-type Columbia; *fad2*, *FAD2* mutant; *fad2/FAD2-1* and *fad2/FAD2-2*, two transgenic *fad2* lines complemented with the functional Col-0 *FAD2* gene; *fad2/FAD2Δ-1* and *fad2/FAD2Δ-2*, two transgenic *fad2* lines complemented with the mutated *FAD2Δ* gene. Results are presented as means and standard errors from three independent experiments. ** indicates significant difference in comparison to Col-0 at P<0.01 (Student's t-test).

To dissect the function of *FAD2*, we obtained a *fad2* mutant from the Arabidopsis Biological Resource Centre (CS8041). Sequence analysis indicated that the *fad2* allele harbored a site mutation in the second exon, 310 bp downstream of ATG (Fig. 3A), leading to an amino acid mutation (A104T) adjacent to the first highly conserved His-box (105-110: HECGHH), which and the other two His-boxes coordinate the iron atoms at the active site center (Fig. 3B). Fatty acid composition analyses revealed that the levels of monounsaturated fatty acids (18∶1) increased significantly in *fad2*, whereas the levels of polyunsaturated fatty acids (18∶2 and 18∶3) decreased significantly, when compared with those in the wild-type plants. Complementary expression of the functional *FAD2* gene (ProFAD2:FAD2) from the wild-type plants, but not of the loss-of-function gene (*FAD2Δ*) from *fad2* (ProFAD2:FAD2Δ), controlled by its native promoter, fully recovered the fatty acid composition in *fad2* (Fig. 3C).

### *Fad2* is hypersensitive to salt and osmotic stresses

**Figure 4:**
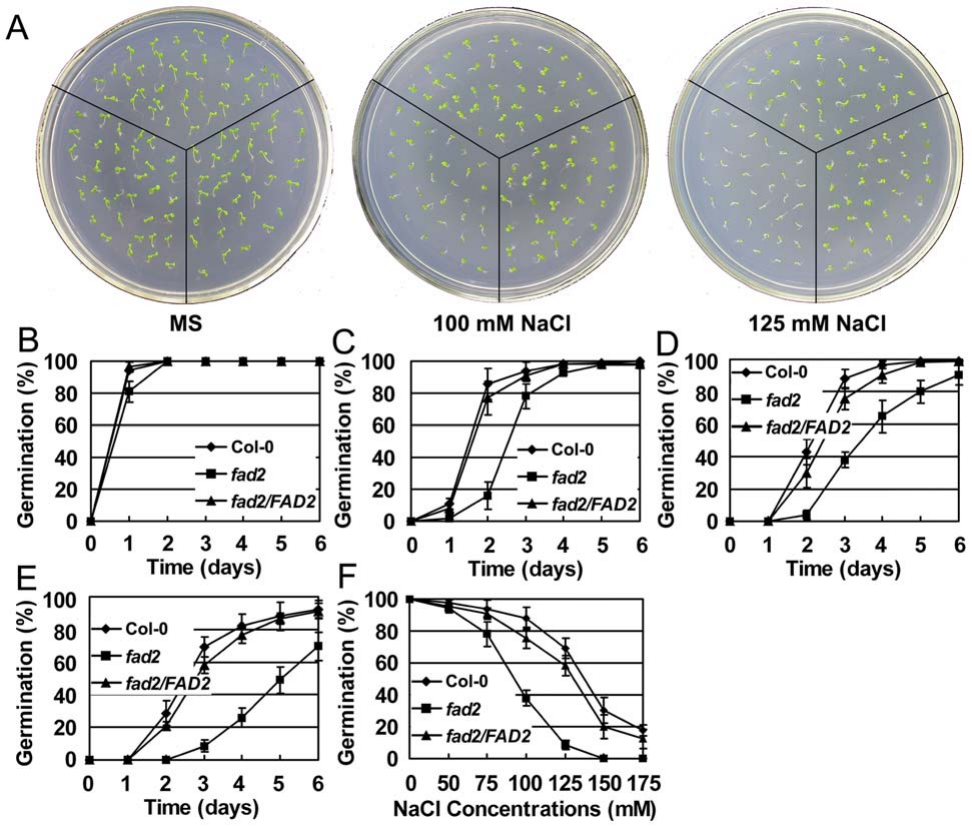
Germination of Col-0, *fad2* and *fad2/FAD2* under salt treatments. (A) Seeds were sowed on MS medium supplemented with 0 (left), 100 (middle) or 125 mM NaCl (right). Photos were taken seven days after stratification. (B)–(E) Percentage of germinating seeds grown on MS medium supplemented with 0 (B), 75 (C), 100 (D) or 125 (E) mM NaCl. F, Percentages of germinating seeds grown on MS medium supplemented with different concentrations of NaCl three days after stratification. Results are presented as means and standard errors from three independent experiments (≥100 seeds of each line were sown for each experiment).

**Figure 5:**
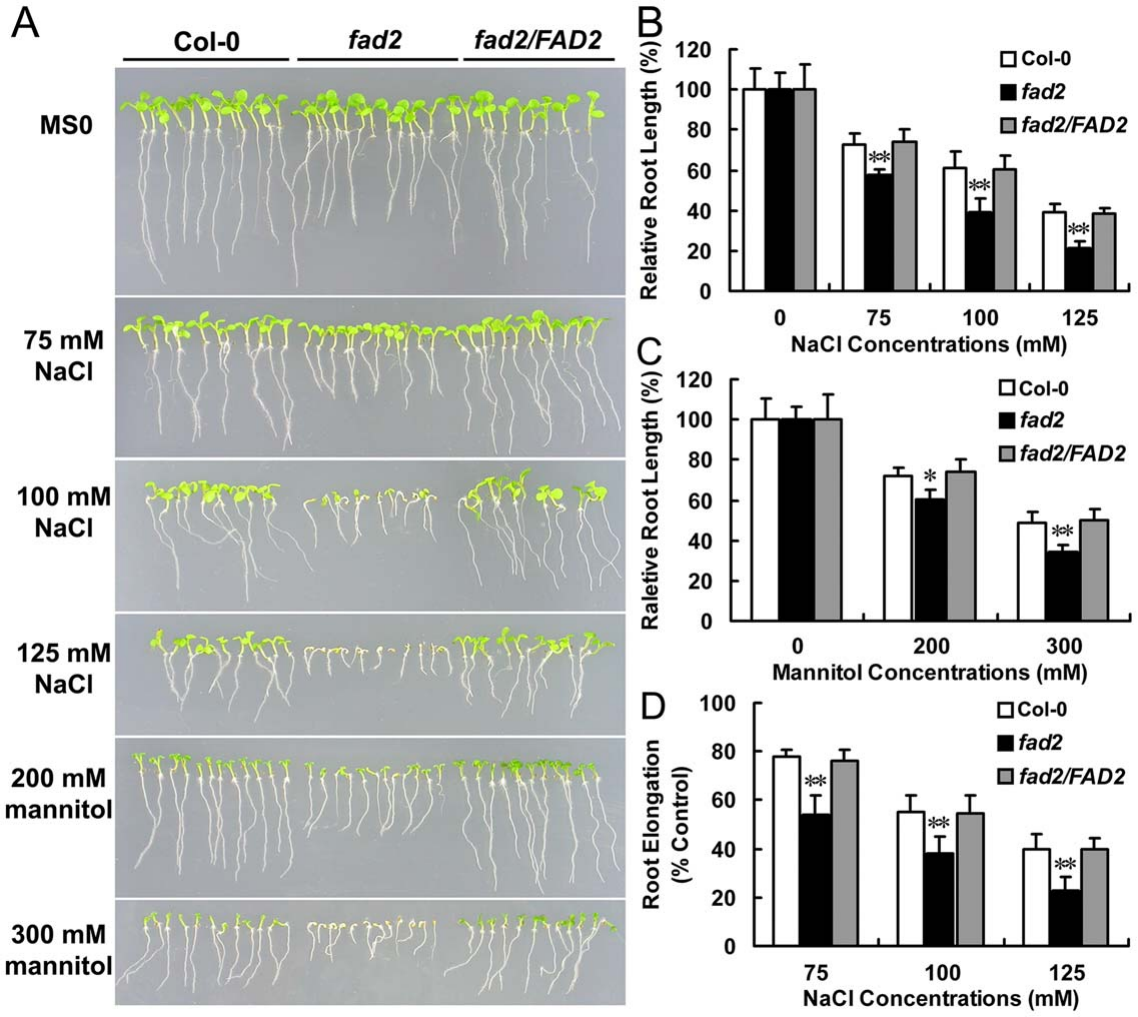
Stress response of Col-0, *fad2* and *fad2/FAD2*. Col-0, wild-type Columbia; *fad2*, *FAD2* mutant; *fad2/FAD*, transgenic *fad2* line 1 complemented with the Col-0 *FAD2* gene (*fad2/FAD2-1*). (A) Phenotypes on MS medium supplemented with different concentrations of NaCl or mannitol. Photos were taken 7 days after the initiation of the treatments. B and C, Root growth on MS medium supplemented with different concentrations of NaCl (B) or mannitol (C) from (A). The relative root lengths were measured on day 7 after stratification (n = 60 for each condition). (D) Root elongations on MS medium supplemented with different concentrations of NaCl. Four-day-old seedlings were transferred to MS medium supplemented with 0, 75, 100 or 125 mM NaCl. Results are presented as means and standard errors from three independent experiments. * and ** indicate significant differences in comparison to Col-0 at P<0.05 and P<0.01, respectively (Student's t-test).

We compared the seed germination, root growth and survival rate of Col-0, *fad2*, and *fad2/FAD2* under different stress conditions. Seeds germinated on MS medium containing different concentrations of NaCl. Germination rates of wild-type and *fad2* were similar when cultured on normal MS medium (Fig. 4A, 4B). However, germination of *fad2* seeds was significantly impaired on MS medium supplemented with 100 or 125 mM NaCl, whereas germination of wild-type seeds was less strongly affected (Fig. 4C–4F). After three days on high salt MS medium (100 mM NaCl), only ∼38% of the *fad2* seeds, but more than 80% of the wild-type seeds germinated (Fig. 4D). When sown on MS medium supplemented with 125 mM NaCl, only ∼12% of the *fad2* seeds, but ∼73% of the wild-type seeds germinated after 3 days (Fig. 4E). To further test the effect of salt on growth, we compared the root growth of *fad2* and wild-type seedlings grown under high salt or osmotic stress condition. Under normal growth condition, root growth of *fad2* is slightly impaired. Therefore relative root growth is examined. Root growth of Col-0, *fad2*, and *fad2/FAD2* on medium supplemented with different concentrations of NaCl or mannitol relative to that on medium without NaCl or mannitol was examined. When grown under high salt or mannitol condition, root growth retardation of *fad2* was greater than that of Col-0 and *fad2/FAD2* at all concentrations of NaCl and mannitol tested (Fig. 5A–5C). Furthermore, in order to eliminate the inhibition of NaCl on germination, we also tested the root growth of Col-0, *fad2*, and *fad2/FAD2* after germination. Four-day-old seedlings were transferred to MS medium plates supplemented with or without NaCl, and vertically cultured for six days. The root elongation of *fad2* was also less than that of Col-0 and *fad2/FAD2* (Fig. 5D). The greater root growth inhibition of *fad2* indicated that under the imposed salt or mannitol stress conditions, *fad2* is more sensitive to the stress treatment. The sensitivity of *fad2* salt or osmotic stress was recovered by expression of *FAD2* in *fad2* mutant (Fig. 4 and Fig. 5).

**Figure 6:**
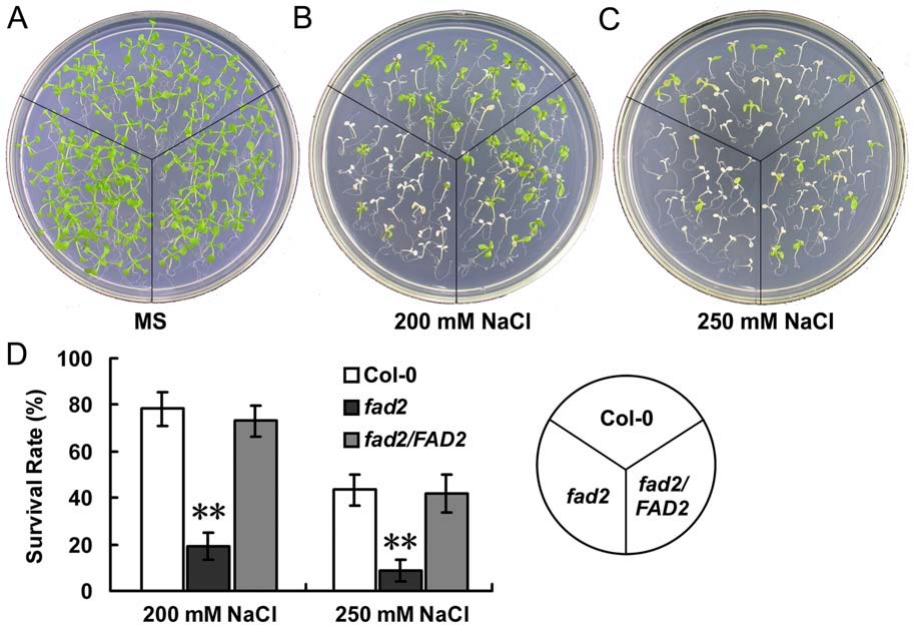
Survival rrates of Col-0, *fad2* and *fad2/FAD2*. (A)-(C), Five-day-old seedlings of Col-0, *fad2* and *fad2/FAD2* (*fad2/FAD2-1*) were transferred to MS medium supplemented with 0 (A), 200 (B), or 250 mM NaCl (C). Photos were taken 10 days after the initiation of the stress treatments. (n = 240). (D) Survival rates of plants in (B) and (C). Results are presented as means and standard errors from three independent experiments. ** indicates significant difference in comparison to wild-type at P<0.01 (Student's t-test) (n = 240).

To test whether *fad2* can survive high salt stress, we also quantified the survival rates of Col-0, *fad2* and *fad2/FAD2* grown under high salt stress condition. Five-day-old seedlings were transferred to MS medium plate supplemented with 0, 200 or 250 mM NaCl, and grown for ten days under short day condition. The survival rates of wild-type and *fad2* seedlings were similar when cultured on normal MS medium (Fig. 6A). However, the survival rate of *fad2* was much lower on MS medium supplemented with 200 or 250 mM NaCl, whereas the survival rate of wild-type was less strongly affected: after ten days on high salt MS medium (250 mM NaCl), only ∼10% of the *fad2* seedlings, but almost 42% of the wild-type seedlings survived (Fig. 6B–6D). Again, the salt sensitive phenotypes of *fad2* were recovered by expression of *FAD2* in *fad2* mutant (Fig. 6B-6D).

### *Fad2* accumulated more Na^+^ and less K^+^ under high salt condition

**Figure 7:**
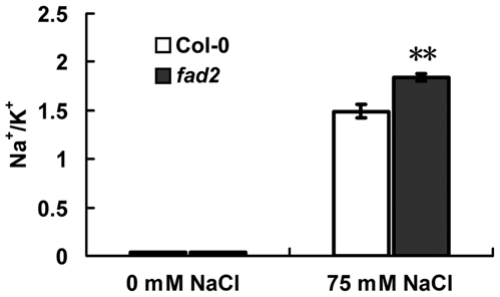
Na^+^/K^+^ Ratio analyses in Col-0 and *fad2* mutant. Results are presented as means and standard errors from three independent experiments. ** indicates significant difference in comparison to Col-0 at P<0.01 (Student's t-test). Eight-day-old seedlings grown on MS medium supplemented with or without 75 mM NaCl were used.

It is well known that cytosolic Na^+^/K^+^ ratio is a key determinant of plant salinity tolerance. The Na^+^ and K^+^ contents in wild-type and *fad2* plants were examined. When the seedlings were grown in the absence of NaCl, no significant differences were seen in the Na^+^ and K^+^ contents of wild-type and mutant plants (Fig. 7). Whereas, upon the treatment with 75 mM NaCl, *fad2* accumulated less K^+^ and substantially more Na^+^, leading to an increased Na^+^/K^+^ ratio in the mutant (Fig. 7), suggesting the possible involvement of fatty acid unsaturation in the regulation of Na^+^ and other ion homeostasis under salt stress condition.

### Increased malondialdehyde (MDA) accumulation in *fad2*

Changes in the levels of lipid hydroperoxide accumulation induced by salt stress were also measured by determining MDA content. Upon treatment with 75 mM NaCl, MDA concentration markedly increased in *fad2* plants, whereas only a marginal increase was detected in the wild-type plants (Fig. S1A).

### Superoxide dismutase (SOD) and catalase (CAT) activities were affected in *fad2*

Lipid hydroperoxidation is an effective indicator of cellular oxidative damage [22]. Although the activity of ascorbate peroxidase (APX) was largely unaffected in wild-type and *fad2* plants, significant decrease of SOD and CAT activities was observed in the *fad2* mutant after treatment with 75 mM NaCl (Fig. S1B). These results indicate that disruption of FAD2 function reduced the tolerance to salt stress-induced membrane hydroperoxidation in *fad2* plants.

### Reduced polyunsaturated fatty acid composition in the membrane lipids isolated from *fad2*

**Figure 8:**
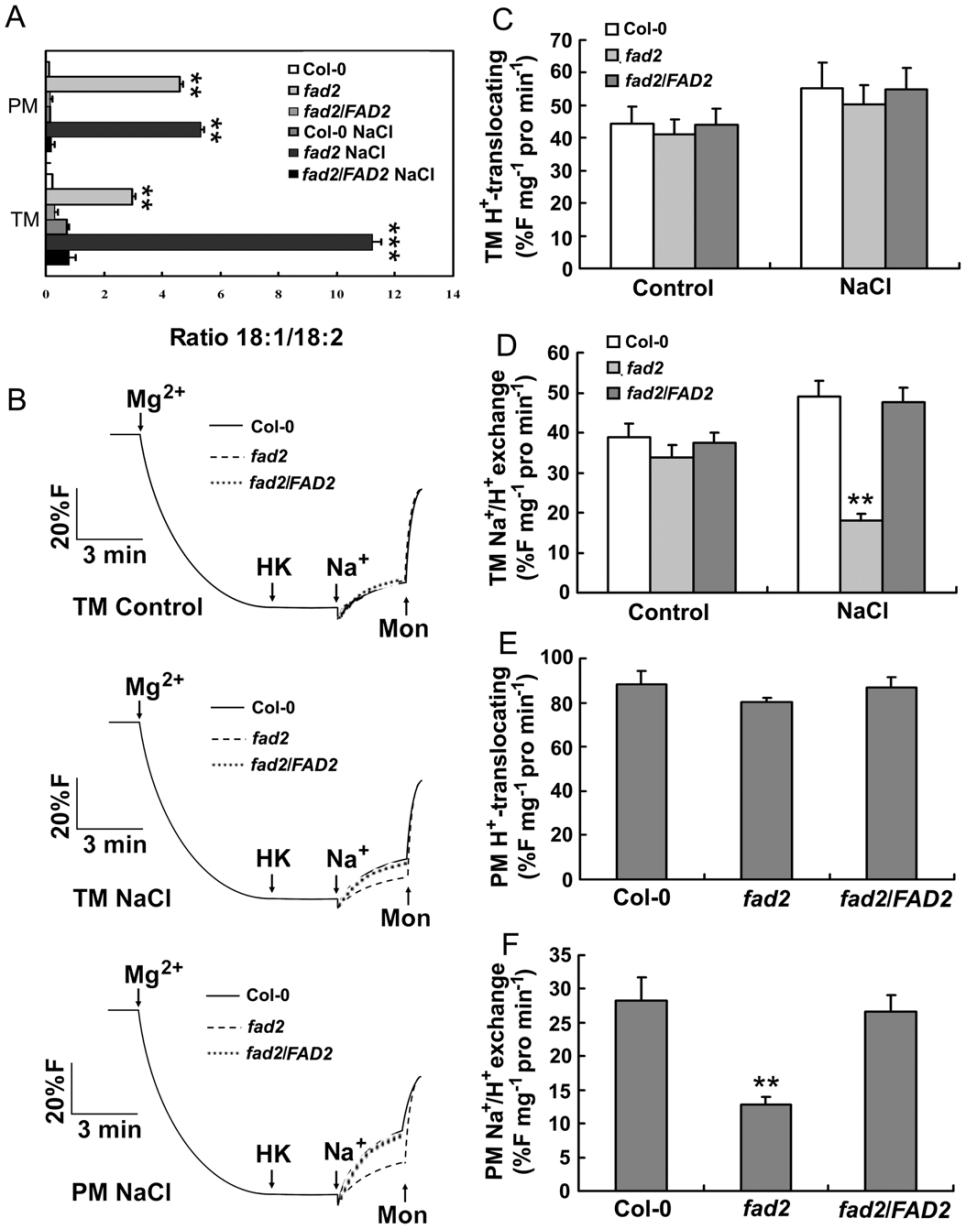
Fatty acid composition and Na^+^/H^+^ exchanger activity assays. (A) Fatty acid compositions. (B) Na^+^-dependent H^+^ exchange. TM control, tonoplast vesicles without NaCl treatment (upper); TM NaCl, tonoplast vesicles with NaCl treatment (middle); PM NaCl, plasma membrane vesicles with NaCl treatment (lower). (C) and (E) Rates of H^+^ translocation of tonoplast (C) and plasma membrane (E) vesicles. (D) and (F) Rates of Na^+^-dependent H^+^ effluxes in tonoplast (D) and plasma membrane (F) vesicles. Tonoplast and plasma membrane vesicles were isolated from the leaves of 4-week-old seedlings treated with or without 250 mM NaCl for 3 days. Col-0, wild-type Columbia; *fad2*, *FAD2* mutant; *fad2/FAD*, *fad2/FAD2-1.* Results are presented as means and standard errors from four independent experiments. ** and ** indicate significant difference in comparison to wild-type at P<0.01 and P<0.001 (Student's t-test).

To understand whether the reduced polyunsaturated fatty acid composition in *fad2* decreased the fluidization of membrane lipids, we analyzed the 18∶1 and 18∶2 content of vacuolar (tonoplast) and plasma membrane isolated from leaves of 4-week-old seedlings of Col-0, *fad2* and *fad2/FAD2* grown under normal or salt stress conditions. Consistent with the total fatty acid analyses, under either condition, both kinds of membranes isolated from *fad2* contained a lower level of 18∶2 and a higher level of 18∶1, when compared with Col-0 and *fad2/FAD2* plants (Fig. 8A).

### Decreased vacuolar and plasma Na^+^/H^+^ exchange activity in *fad2*

To determine whether the altered fatty acid unsaturation of vacuolar and plasma membrane would affect the Na^+^/H^+^ exchange activity, Na^+^-dependent H^+^ movements were measured in tonoplast [23] and plasma membrane [24] vesicles isolated from the leaves of Col-0, *fad2* and *fad2/FAD2*. The Na^+^/H^+^ exchange rates were very low in tonoplast and plasma membrane vesicles from *fad2* mutant (Fig. 8B–8F). In contrast, Na^+^/H^+^ exchange rates were much higher in vesicles isolated from the leaves of Col-0 and *fad2/FAD2* (Fig. 8B-8F).

### Altered subcellular Na^+^ distribution in *fad2*

**Figure 9:**
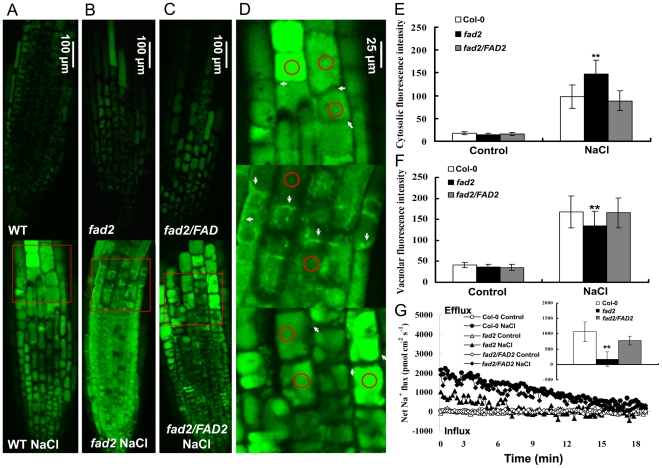
Intracellular Na^+^ distribution and Na^+^ extrusion in roots of Col-0, *fad2* and *fad2/FAD2*. (A)-(C) Representative examples (n = 10–12) of CoroNa-Green staining of the root tip region before and after NaCl treatment. (D) Magnifications of marked regions (red boxes) in (A) to (C). Red circles and white arrows indicate the vacuolar and cytoplamic regions, respectively. (E) and (F) Fluorescent intensities in the vacuole and cytosol were calculated for comparison. Error bars indicate SD of at least 100 cells randomly measured from 10 individual seedlings. (G) Net Na^+^ efflux in root tips. Inserted section show the mean flux rates within the measuring period of 18–20 min in NaCl-treated seedlings. ** indicates significant difference in comparison to wild-type at *p*<0.01 (Student's t-test). Roots of five-day-old seedlings were stained with CoroNa-Green AM after 72 h of 100 mM NaCl treatment and observed with a confocal microscope.

To understand how the decreased Na^+^/H^+^ exchanger activity would affect the sodium distribution in *fad2*, we examined the Na^+^ compartmentalization and extrusion by CoroNa-Green staining. We observed that Na^+^ was compartmented into vacuoles of root cells in Col-0 and *fad2/FAD2* seedlings, whereas Na^+^ was accumulated in the cytoplasm of root cells in *fad2* mutant (Fig. 9A–9F). Net Na^+^ efflux analysis in root tips of Col-0, *fad2* and *fad2/FAD2* seedlings further demonstrated that Na^+^ efflux was also lower in *fad2* than that in Col-0 and *fad2/FAD2* (Fig. 9G).

## Discussion

When plants are subject to adverse environmental conditions, a wide range of cellular response occurs, including the adjustments of unsaturation levels of membrane fatty acids. Mutants of Arabidopsis that contain reduced levels of polyunsaturated fatty acids were more sensitive to low temperature [11]. Here, we demonstrate that fatty acid desaturation by FAD2 is required for salt tolerance in Arabidopsis.

Environmental stresses such as cold, heat, drought and salt induce changes in FA composition. Many fatty acid desaturases were involved in this process. Expression of *FAD8* was strongly induced by low temperature in Arabidopsis [2], and expression of *FAD6* was responsive to salt and osmotic stress [16]. In order to investigate the physiological effects of fatty acid unsaturation on plants growth under salt stress, we examined the expression pattern of *FAD2* in Arabidopsis, and compared the growth feature of wild-type with that of *fad2*, an Arabidopsis mutant which contains increased levels of monounsaturated fatty acids and reduced levels of dienoic fatty acids. RT-PCR analysis of *Arabidopsis thaliana* Columbia (Col-0) and β-Glucuronidase (GUS) staining of transgenic plants carrying the native *FAD2* promoter *GUS* reporter gene (ProFAD2:GUS) in Col-0 indicated that *FAD2* transcripts were ubiquitously present in seedlings and various tissues (Fig. 1A–1C). This is consistent with the microarray data obtained from the Genevestigator (https://www.genevestigator.com)(Fig. S2). Further analyses showed that the expression of *FAD2* is regulated by adverse environmental factors (Fig. 1D), suggesting the possible involvement of *FAD2* in plant response to abiotic stress.

Transgenic tobacco plants expressing the Arabidopsis antisense *FAD7* DNA fragment contained reduced levels of polyunsaturated fatty acids, and were more sensitive to drought/salt stress [9]. On the contrary, heterologous expression of sunflower (*Helianthus annuus*) oleate Δ12 desaturases (*HaFAD2*-*1* or *HaFAD2*-*3*) in *Saccharomyces cerevisiae* increased the content of dienoic fatty acids (18∶2), and thereby increased the tolerance of yeast cells to NaCl [21]. Expression of the functional *FAD2*, but not *FAD2*Δ, also increased the tolerance of yeast cells to salt (Fig. 2).

The Arabidopsis *FAD2* mutant (*fad2*) is deficient in activity of FAD2. The amount of 18∶2 and 18∶3 in *fad2* was lower than that in Col-0 (Fig. 3C). This reduction in polyunsaturated fatty acid content was associated with an increase of the corresponding 18∶1 precursor (Fig. 3C). Long-term culture of *fad2* plants at low temperature (6°C) eventually resulted in their withering and death, but not for wild-type plants [25]. Besides functions on cold and heat tolerance, polyunsaturated fatty acids also may have important function on salt tolerance in plant. To understand whether decreased polyunsaturated fatty acid composition in *fad2* would affect its resistance to high salinity, we performed salt tolerance analyses. Interestingly, *fad2* is much more sensitive to NaCl than the previously reported *fad6* mutant (Fig. S3). All these results demonstrate that *FAD2* is required for salt tolerance in Arabidopsis.

Increased production of 18∶3 has been found to accompany cold acclimation in many plants [26], and a positive relationship was also observed between a higher degree of fatty acid desaturation and both cold and freezing tolerance [27]. Both salt and drought stress were found to reduce the amount of 18∶3 [10].

On the basis that yeast cells expressing *FAD2* were more salt tolerant (Fig 2), and *fad2* was hypersensitive to salt and accumulated more sodium (Fig. 4, 5, 6, 7), we hypothesized that the reduced polyunsaturated fatty acid composition in *fad2* decreased the fluidization of member lipids, and consequently impaired the Na^+^/H^+^ exchangers' activity localized on the vacuolar and plasma membrane. In order to testify this hypothesis, we analyzed the 18∶1 and 18∶2 contents of vacuolar (tonoplast) and plasma membrane isolated from Col-0, *fad2* and *fad2/FAD2.* Under either normal or salt stress condition, both kinds of membranes isolated from *fad2* contained a lower level of 18∶2 and a higher level of 18∶1, when compared with Col-0 and *fad2/FAD2* plants (Fig. 8A), suggesting that FAD2 plays an important role in regulating the fatty acid composition of intracellular membrane lipids.

Cell membranes serve as a barrier to the passage of most ions and large molecules, owing to the hydrophobic interior of the lipids. Membrane integrity and function, determined by structure and fluidity, are largely affected by lipid composition and the degree of fatty acid desaturation in plants and other organisms [28]. Researches on *Cyanobacteria* and *Saccharomyces cerevisiae* indicated that fatty acid unsaturation is important for salt stress. *Cyanobacteria* is considered an analogy to plant chloroplasts. Mutants of the *Synechocystis* which lack *ω*-6 and *ω*-3 desaturase activities (desA^-^/desD^-^) contain monounsaturated fatty acids only. Tolerance to and recovery of the photosynthetic machinery of these mutants from salt stress was much reduced compared to the wild-type which contained polyunsaturated fatty acids [29].

Maintenance of ion homeostasis, especially maintenance of a low cytosolic Na^+^/K^+^ concentration ratio, is a key requirement for plant growth in high salt. To maintain a low Na^+^/K^+^ ratio in the cytosol, plants could extrude Na^+^ out of cell using plasma membrane Na^+^/H^+^ antiporters and compartmentate Na^+^ into vacuolar using vacuolar Na^+^/H^+^ antiporters (NHXs) [30], [31]. The vacuolar Na^+^/H^+^ antiporters transport Na^+^ into the vacuole by using the electrochemical gradient of protons generated by the vacuolar H^+^-translocating enzymes, H^+^-adenosine triphosphatase (V-ATPase) and H^+^-inorganic pyrophosphatase (V-PPase) [23], [32]. The plasma membrane Na^+^/H^+^ antiporters extrude Na^+^ out of cell by using the electrochemical gradient of protons generated by the plasma membrane proton pump ATPase (PM-ATPase) [33].

Unsaturated fatty acids play an essential role in the biophysical characteristics of cell membranes, and determine the proper function of membrane-attached proteins [34], [35]. To understand whether the decreased fatty acid unsaturation of vacuolar and plasma membrane would affect the Na^+^/H^+^ exchangers' activity, we isolated tonoplast [23] and plasma membrane [31] vesicles from the leaves of Col-0, *fad2* and *fad2/FAD2*. As we have expected, the Na^+^/H^+^ exchange rates were very low in tonoplast and plasma membrane vesicles from *fad2* mutant (Fig. 8B-8F). In contrast, Na^+^/H^+^ exchange rates were much higher in vesicles isolated from the leaves of Col-0 and *fad2/FAD2* (Fig. 8B-8F). Moreover, the proton translocating activity of V-ATPase, V-PPase and PM-ATPase was also lower in *fad2* mutant than that in Col-0 and *fad2/FAD2* (Fig. S4A-S4C). However, no significant changes were detected in the transcripts of *AtAHA1*, *AtAVP1*, as well as of the genes encoding the plasma membrane (*AtSOS1*) and the vacuolar (*AtNHX1*, *AtNHX2* and *AtNHX5*) Na^+^/H^+^ antiporters (Fig. S4D). These results suggest that the function of Na^+^/H^+^ antiporters were repressed in *fad2* mutant under salt stress growth condition.

Maintaining low levels of sodium ions in the cell cytosol is critical for plant growth and development. The compartmentation of Na^+^ into the plant vacuoles provides an efficient mechanism to avoid the toxic effects of Na^+^ ion in the cytosol [32]. The Na^+^/H^+^ exchangers in the plasma membrane of plant cells also contribute to cellular sodium homeostasis by transporting sodium ions out of the cell [24]. Sodium accumulation in the halophytic Arabidopsis-relative *Thellungiella salsuginea* has been determined in comparison with Arabidopsis [36]. Arabidopsis plants accumulated more Na^+^ than *Thellungiella* [36]. The staining by CoroNa-Green indicated that, Col-0 and *fad2/FAD2* seedlings compartmented Na^+^ into vacuoles, whereas *fad2* mutant failed to do so and accumulated Na^+^ in the cytoplasm of root cells (Fig. 9A-9F). Furthermore, Net Na^+^ efflux was much lower in *fad2* than that in Col-0 and *fad2/FAD2* (Fig. 9G). These are consistent with the decreased Na^+^/H^+^ exchange rates in tonoplast and plasma membrane vesicles isolated from *fad2* (Fig. 8B-8F).

Previously, overexpression of either *FAD3* or *FAD8* increased tolerance to drought in tobacco plants [10]. To see whether overexpression of *FAD2* would increase the salt tolerance in Arabidopsis, we generated transgenic Col-0 plants expressing *FAD2* under the control of two copies of the cauliflower mosaic virus 35S promoter. Surprisingly, transgenic plants were not more salt tolerant than Col-0, as we had expected (Fig. S5). This may be due to the abundant expression of the native *FAD2* (Fig. 1A and Fig. S2) and the limited supply of 18∶1 substrate in Col-0 (Fig. 3C and Fig. S6).

To date, limited information is available concerning the molecular events that take place during fatty acid desaturation upon salt stress in Arabidopsis other than the fact that fatty acid desaturation is important for temperature acclimation [9]. Our results suggest that FAD2-mediated fatty acid desaturation of vacuolar and plasma membrane is required for proper sodium extrusion and vacuolar compartmentation, and thereby mediates salt tolerance in Arabidopsis.

## Materials and Methods

### Plant materials and RNA isolation

Wild-type *Arabidopsis thaliana* (ecotype Columbia-0), and mutant *fad2* (CS8041, Arabidopsis Biological Resource Centre) were grown in the green house as described previously [37], [38]. The *fad2* mutant was backcrossed to wild-type Arabidopsis (Col-0) twice, then homologous line was used for stresses assay. For NaCl or mannitol treatment, 300 mM NaCl or 300 mM mannitol was added to Murashige and Skoog (MS) plates [39], and 8-day-old seedlings were incubated under normal growth condition. Total RNA was isolated with the TRIZOL Reagent (Invitrogen, Shanghai, China) following the manufacturer's instruction. For analyses of tissue expression of *FAD2*, total RNA was extracted from various tissues of Col-0 plants, and treated with RNase-free DNase (Promega, Shanghai, China). The first strand cDNA synthesis was performed with the ReverTra Ace kit (TOYOBO, Shanghai, China).

### Reverse transcriptase-PCR and quantitative real-time PCR analyses

Reverse transcriptase-mediated PCR (RT-PCR) was performed using *FAD2* forward (5′-CCAAAGCAGAAATCAGCAATCA-3′) and reverse (5′-GCAGCAGCGTAACGG TAAAGAC-3) primers to amplify a PCR product of 260 bp. Expression level of *ACTIN2* (At3g18780) was also determined with forward (5′-CATCCTCCGTCTTGAC CTTGC-3′) and reverse (5′-CA AACGAGGGCTGGAACAAG-3′) primers (to serve as a quantitative control). For RT-PCR analyses of *AtAHA1, AtSOS1, AtAVP1, AtNHX1, AtNHX2* and *AtNHX5*, gene specific primers were used (Table S1). For quantitative real-time PCR, cDNA was synthesized, and DNA amplification was performed in the presence of SYBR Green Realtime PCR Master Mix (QPK-201) (TOYOBO) on the Rotor-Gene real-time thermocycler R3000 (Corbett Research) using the same primer pairs as in the RT-PCR. The relative mRNA levels of *FAD2* were determined by normalizing the PCR threshold cycle number with that of *ACTIN2* [40]. All experiments were repeated three times independently, and the average was calculated.

### FAD2 promoter-β-g**alactosidase** (GUS) expression in transgenic Arabidopsis plants

To generate the *FAD2* promoter-GUS construct (ProFAD2:GUS), the 5′-flanking DNA of the *FAD2* coding region was amplified with *FAD2* specific primers (forward: 5′-CTGAATATGTTGGAGTCTTTGCTA-3′; reverse: 5′-GTTTCTGCAGAAAACCA AAAGC-3′). The 2411 bp PCR fragment was cloned into the pCAMBIA1300+pBI101 vector for sequence confirmation [41]. Then the construct was transformed into Col-0 plants as described previously [42]. For histochemical analyses, materials were stained with 5-bromo-4-chloro-3-indolyl-D-glucuronide at 37°C for 6 h, followed by incubation in 75% ethanol [43].

### *fad2* mutant identification and complementation

The genomic DNA fragment containing the whole *FAD2* gene was amplified from Col-0 and *fad2* mutant using KOD-Plus DNA polymerase and *FAD2* specific primers (forward: 5′-CTGAATATGTTGGAGTCTTTGCTA-3′; reverse: 5′-GTTTTGTCCCTCTTATACA CTTGG-3′). The PCR product was cloned into pBCKS vector via *Eco*R V site. Three clones were selected for sequence confirmation of the mutation site. The whole *FAD2* fragment containing the *FAD2* promoter, ORF and 3′ -UTR amplified from Col-0 or *fad2* mutant was ligated into pHB plant expression vector [44]. The construct (ProFAD2:FAD2 or ProFAD2:FAD2Δ) was transformed into *fad2* mutant [42], and transformants were selected on 30 mg/L hygromycin.

### Total fatty acid analyses

Total fatty acids (FAs) were extracted from 8-day-old seedlings (0.5 g) as described previously [45]. Direct transesterification was made by the addition of 1 ml 3% H_2_SO_4_ in methanol and incubating at 100°C for 3 h. Methylated FAs were determined by gas-liquid chromatography using a model Agilent 19091S-433 gas-liquid chromatograph. The commercial standard FAME mixture (SUPELCO, USA) was estimated quantitatively using methylnonadecanoate as an internal standard [10].

### Heterologous expression of FAD2 in yeast

The ORF of *FAD2* or *FAD2*Δ (the mutation allele in *fad2* mutant) was amplified from Col-0 and *fad2* mutant using *FAD2* specific primers (forward: 5′-CATCTCCAGAAAC ATGGGTGC-3′; reverse: 5′-TTCTTCACCATCATCCTCATAACT-3′) and cloned into *pVT102-U* [46] to produce a yeast expression vector *pVTFAD2* or *pVTFAD2*Δ. After sequencing confirmation, the plastids were transformed into yeast strain W303-1 using LiAC method described in the Yeast Protocols Handbook (Clontech, USA). *pVTHaFAD2-1* and *pVTHaFAD2-3* [21], which contain the sunflower (*Helianthus annuus*) genes *HaFAD2*-*1* (GenBank accession number AF251842) and *HaFAD2*-*3* (GenBank accession number AF251844), respectively, served as positive controls. W303-1 and transformants were grown in SD liquid medium at 30°C until early exponential phase and were diluted to an OD600 of 0.3. Serial 10-fold dilutions (to 10^−3^) of the adjusted cultures (1.5 µl) were spotted onto YPD (1% yeast extract, 2% peptone, 2% glucose) agar solid medium containing different concentrations of NaCl. Colony growth was inspected after 2 to 5 days of incubation at 30°C.

### Germination rate, root growth and survival rate analyses

For germination analyses, approximately 100 surface-sterilized seeds of Col-0, *fad2* mutant and complementary line (*fad2/FAD2*) were planted on MS medium supplemented with different concentrations of NaCl, and incubated at 4°C for 2 days before being placed at 22°C under long-day conditions. Germination was scored six consecutive days, and was considered to have germinated when the radicles penetrated the seed coats. For root growth measurements, 12 seeds each from the Col-0, *fad2* and *fad2/FAD2* line were planted on MS medium supplemented without (MS0) or with different concentrations of NaCl or mannitol, and incubated at 4°C for 2 days before being placed vertically at 22°C under long-day conditions. The root length was measured at day 7 as described previously [47]. The relative root growth was calculated as follows: Root length on MS medium containing different NaCl or mannitol/the average of root length on MS0 medium × 100%. For root elongation measurement after germination, four-day-old seedlings grew vertically on MS medium plates were transferred to MS medium plates supplemented without (MS0) or with different concentrations of NaCl, and vertically cultured for six days. Then the root growth during these six days was measured. The root elongation was calculated as follows: Root growth on MS medium containing NaCl/the average of root growth on MS0 medium × 100%. For survival rate analyses, 5-day-old seedlings (80 seedlings of each line under each condition) were transferred individually to MS medium plate or MS medium plate supplemented with 200 or 250 mM NaCl for ten days, then the survival rates were scored. Each experiment was repeated at least three times.

### Na^+^ and K^+^ content determination

Eight-day-old seedlings grown on MS medium supplemented without or with 75 mM NaCl were used for cation content determination with an atomic absorption spectrophotometer as described previously [48] (Z-8000; Hitachi, Tokyo, Japan).

### MDA, SOD, APX and CAT activity analyses

Eight-day-old seedlings grown on MS medium supplemented without or with 75 mM NaCl were used for MDA content, SOD, APX or CAT enzyme activity analyses as described previously [49].

### Membrane isolation, Na^+^/H^+^ exchange activity and fatty acid analyses

Membrane fractions were isolated from young leaves of 4-week-old plants treated with or without 250 mM NaCl for 3 days as described previously [23], [24]. For fatty acids analyses, total fatty acids of tonoplast or plasma membrane was extracted and analyzed as described above. The membrane identity and transport competence of the vesicles were assessed with measurements of the H^+^-transport activity of the plasma membrane H^+^-ATPase or tonoplast H^+^-ATPase and H^+^-PPase activities. The initial rate of fluorescence quenching (percent quenching min^−1^) was used as a relative estimate of the rate of H^+^ translocation. The rates were normalized to the fluorescence change between the addition of Mg^2+^ and the addition of Hexokinase (HK). The Na^+^/H^+^ exchange activity was measured by following the pH-dependent fluorescence quenching of quinacrine as described [23], [24]. These initial rates were taken within the first 3 min after the addition of 50 mM NaCl.

### Visualization of intracellular Na^+^ distributions

To visualize the Na^+^ distributions in the root cells of Col-0, *fad2* and *fad2/FAD2*, a Na^+^-specific fluorescent dye, CoroNa-Green AM (Invitrogen), was used [50]. Five-day-old seedlings grown on MS medium were transferred to a fresh medium containing with 0 mM (control) or 100 mM NaCl for 72 h. Then the seedlings were washed 2–3 times with distilled water and stained with 20 µM CoroNa-Green AM in the presence of pluronic acid (0.02%, Invitrogen) for 3h. The intracellular Na^+^ fluorescence was visualized by a confocal microscopy (TCS SP5; Leica, Wetzlar, Germany). The confocal settings were as follows: excitation 488 nm, emission 510–530 nm, frame 512×512. The Na^+^-specific fluorescence in the cytosolic and vacuolar compartments were calculated by Image-Pro Plus 6.0 software (Media Cybernetics, Bethesda, USA).

### Net Na^+^ flux measurements

Net fluxes of Na^+^ were measured non-invasively using the SIET system (BIO-001A, Younger USA Sci. & Tech. Corp., Amherst, MA, USA; Applicable Electronics Inc., Forestdale, MA, USA and ScienceWares Inc., East Falmouth, MA, USA) as described previously [51]. In brief, five-day-old seedlings grown on MS medium were transferred to a fresh medium containing with 0 mM (control) or 100 mM NaCl for 72 h. Then the seedlings were washed 4–5 times with redistilled water and transferred to the measuring chamber containing 10 to 15 ml measuring solution [0.5 mM KCl, 0.1 mM CaCl_2_, 0.1 mM MgCl_2_, 0.1 mM NaCl, and 2.5% sucrose, pH 5.5 (adjusted with KOH and HCl)). After the roots were immobilized on the bottom, Na^+^-ion flux measurements were started and the measuring site was 500 to 600 µm from the root apex, in which cytosolic Na^+^ accumulation was higher in *FAD2* than that in Col-0 (Figure 9). The steady-state Na^+^ flux measurements were, as a rule, continuously recorded for 15–18 min because that the concentration gradients of Na^+^ in the measuring solution was undetectable after a prolonged salt relief (>20 min).

### SIET performance

The concentration gradients of the target ions were measured by moving the Na^+^ ion-selective microelectrode between two positions close to the roots in a preset excursion (30 µm for intact roots in our experiment) at a programmable frequency in the range of 0.3 to 0.5 Hz. The electrode is stepped from one position to another in a predefined sampling routine while also being scanned with the three-dimensional microstepper motor manipulator (CMC-4). Pre-pulled and silanised glass micropipettes (2–4 µm aperture, XYPG120-2)(Xuyue Sci. and Tech. Co., Ltd., Beijing, China) were treated with a backfilling solution (Na: 100 mM NaCl;) to a length of ca. 1.0 cm from the tip. Then front-filled with ca. 15-µm columns of selective liquid ion exchange cocktails (LIXs) (Na: Fluka 71178). An Ag/AgCl wire electrode holder (XYEH01-1; Xuyue Sci. and Tech. Co., Ltd., Beijing, China) was inserted in the back of the electrode to make electrical contact with the electrolyte solution. DRIREF-2 (World Precision Instruments) was used as the reference electrode. Na^+^-ion selective electrodes were calibrated prior to flux measurements (Na^+^ was usually 0.1 mM in the measuring buffer for root samples). Only electrodes with Nernstian slopes >50 mV/decade were used in our study. Ion flux was calculated by Fick' law of diffusion: *J* = −*D*(*dc/dx*), where *J* represents the ion flux in the *x* direction, *dc/dx*, the ion concentration gradient, and *D*, the ion diffusion constant in a particular medium. Data and image acquisition, preliminary processing, control of the three-dimensional electrode positioner and stepper-motor-controlled fine focus of the microscope stage were performed with ASET software, part of the SIET system.

## Supporting Information

Figure S1 MDA content and antioxidative enzyme activities in Col-0 and *fad2* mutant. A, MDA content. B, SOD, APX and CAT activity analyses. Results are presented as means and standard errors from three independent experiments. * and ** indicate significant differences in comparison to Col-0 at P<0.05 and P<0.01, respectively (Student's t-test). Eight-day-old seedlings grown on MS medium supplemented with or without 75 mM NaCl were used.
(TIF)

Figure S2 The expression pattern of *FAD2* predicted by Genevestigator (https://www.genevestigator.com).
(TIF)

Figure S3 Stress response of Col-0, *fad2* and *fad6* Mutants. Seeds were sown on MS medium supplemented with different concentrations of NaCl. Photos were taken seven days after stratification, and are representatives of three independent experiments.
(TIF)

Figure S4 V-ATPase, V-PPase, PM-ATPase Activity and RT-PCR analyses of *AtAHA1, AtSOS1, AtAVP1, AtNHX1, AtNHX2* and *AtNHX5*. Young leaves of 4-week-old plants of Col-0, *fad2* and *fad2/FAD2* (*fad2/FAD2-1*) treated with or without 250 mM NaCl for 3 days were used. A-C, Tonoplast and plasma membrane fractions were isolated and enzyme activity assays were performed. D, RT-PCR assays. Total RNA was isolated and RT-PCR was performed with gene specific primers (Table S1). Expression of *ACTIN2* was employed as an internal control.
(TIF)

Figure S5 Stress response of *p2X35S:FAD2* transgenic plants, Col-0, *fad2* and *fad2/FAD2* (*fad2/FAD2-1*). Phenotypes on MS medium supplemented with 100 or 125 mM NaCl. Photos were taken 7 days after the initiation of the treatments, and are representatives of three independent experiments.
(TIF)

Figure S6 *FAD2* expression and fatty acid analyses in wild-type and *p2X35S:FAD2* transgenic plants. A, RT-PCR analyses of *FAD2* transcripts. B, Fatty acid analyses. WT, wild-type Col-0; L1-L6, different *p2X35S:FAD2* transgenic lines.
(TIF)

Table S1 Gene specific primer used in this study.
(DOC)
