# Supplementary figures and images for: Arabidopsis Fatty Acid Desaturase FAD2 Is Required for Salt Tolerance during Seed Germination and Early Seedling Growth
Source: PLoS One. 2012 Jan 18;7(1):e30355. doi: 10.1371/journal.pone.0030355 (PMC3261201; doi:10.1371/journal.pone.0030355)

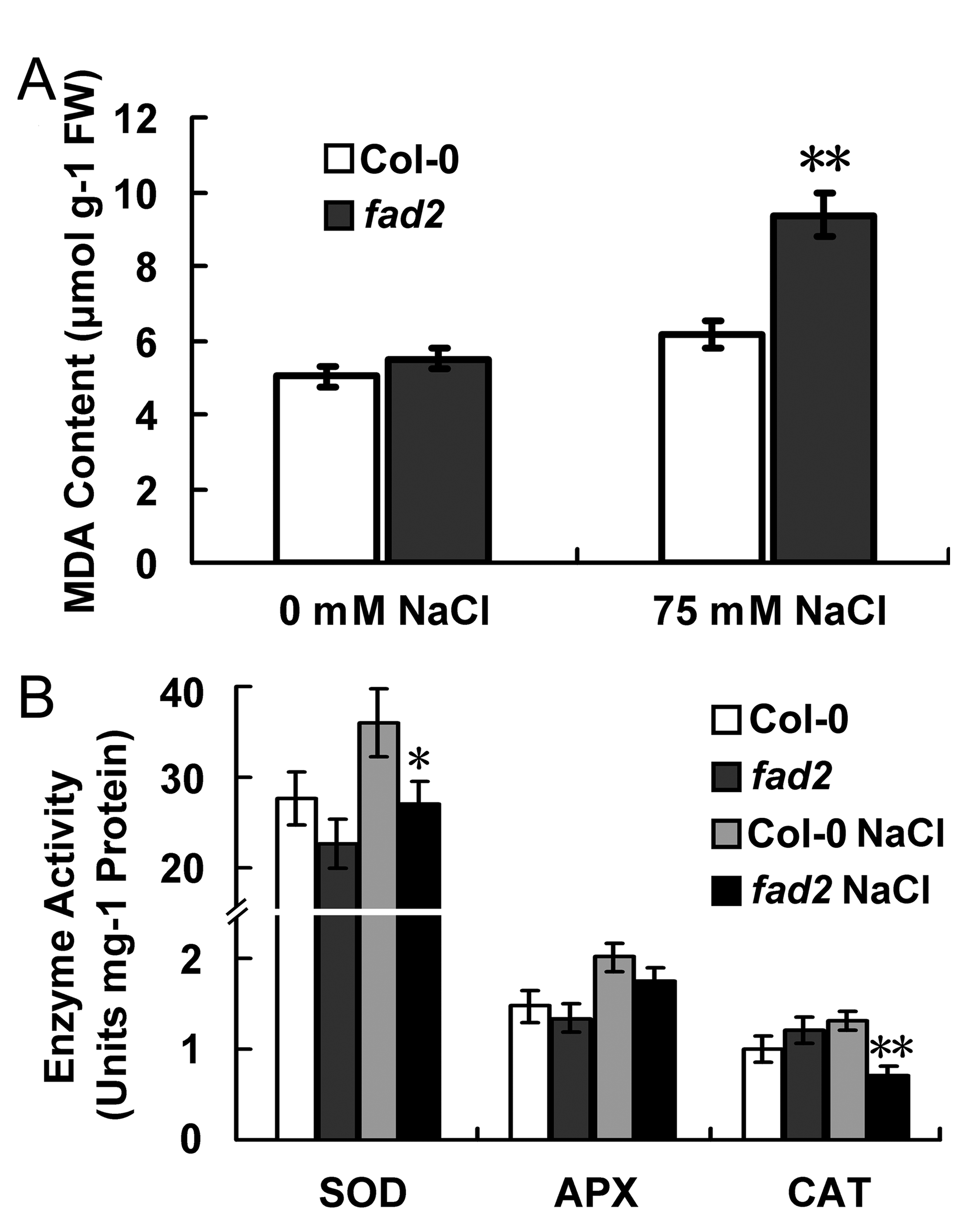

Supplement: Figure S1 — MDA content and antioxidative enzyme activities in Col-0 and fad2 mutant. A, MDA content. B, SOD, APX and CAT activity analyses. Results are presented as means and standard errors from three independent experiments. * and ** indicate significant differences in comparison to Col-0 at P<0.05 and P<0.01, respectively (Student's t-test). Eight-day-old seedlings grown on MS medium supplemented with or without 75 mM NaCl were used. (TIF) [file pone.0030355.s001.tif]

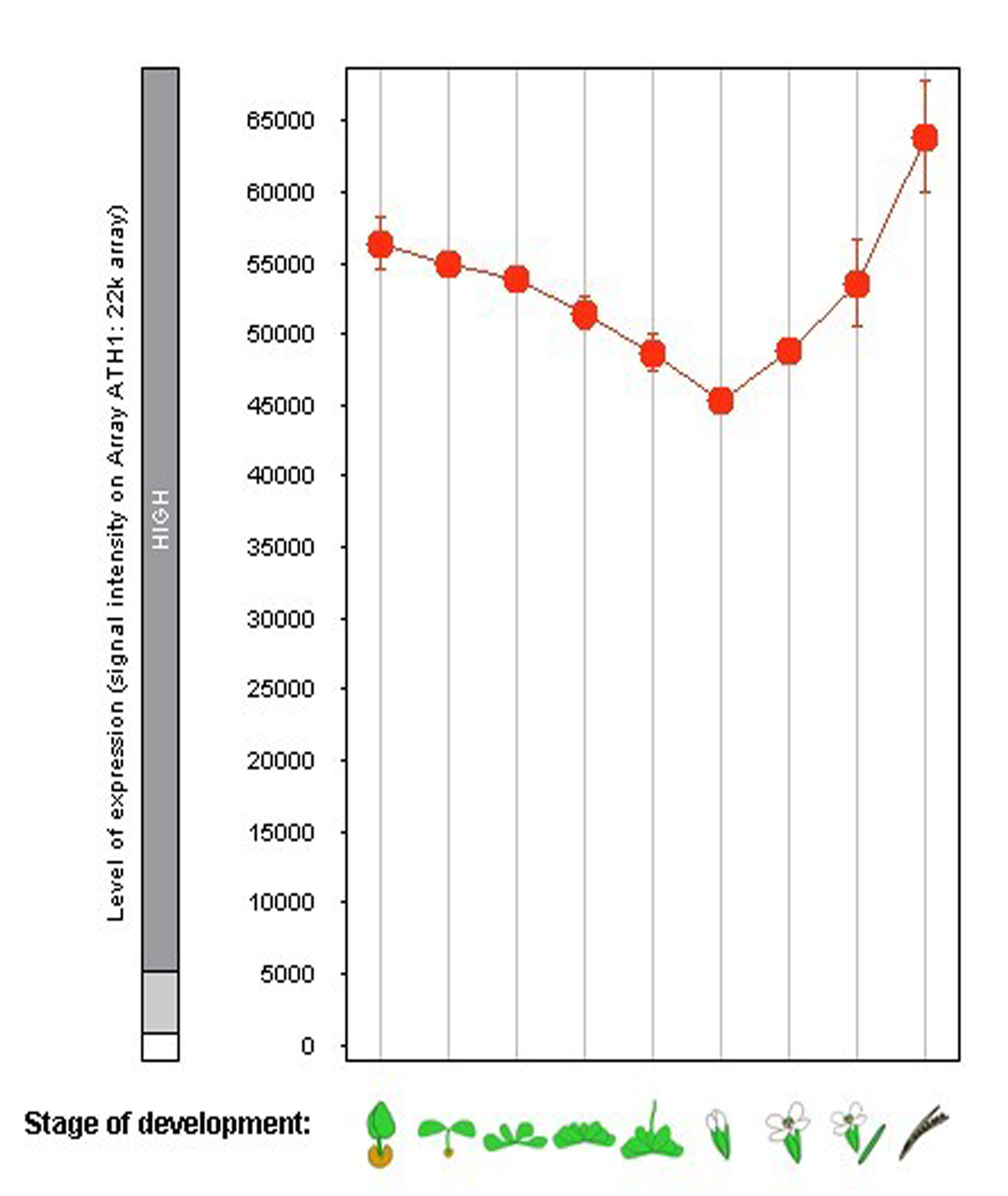

Supplement: Figure S2 — The expression pattern of FAD2 predicted by Genevestigator (https://www.genevestigator.com). (TIF) [file pone.0030355.s002.tif]

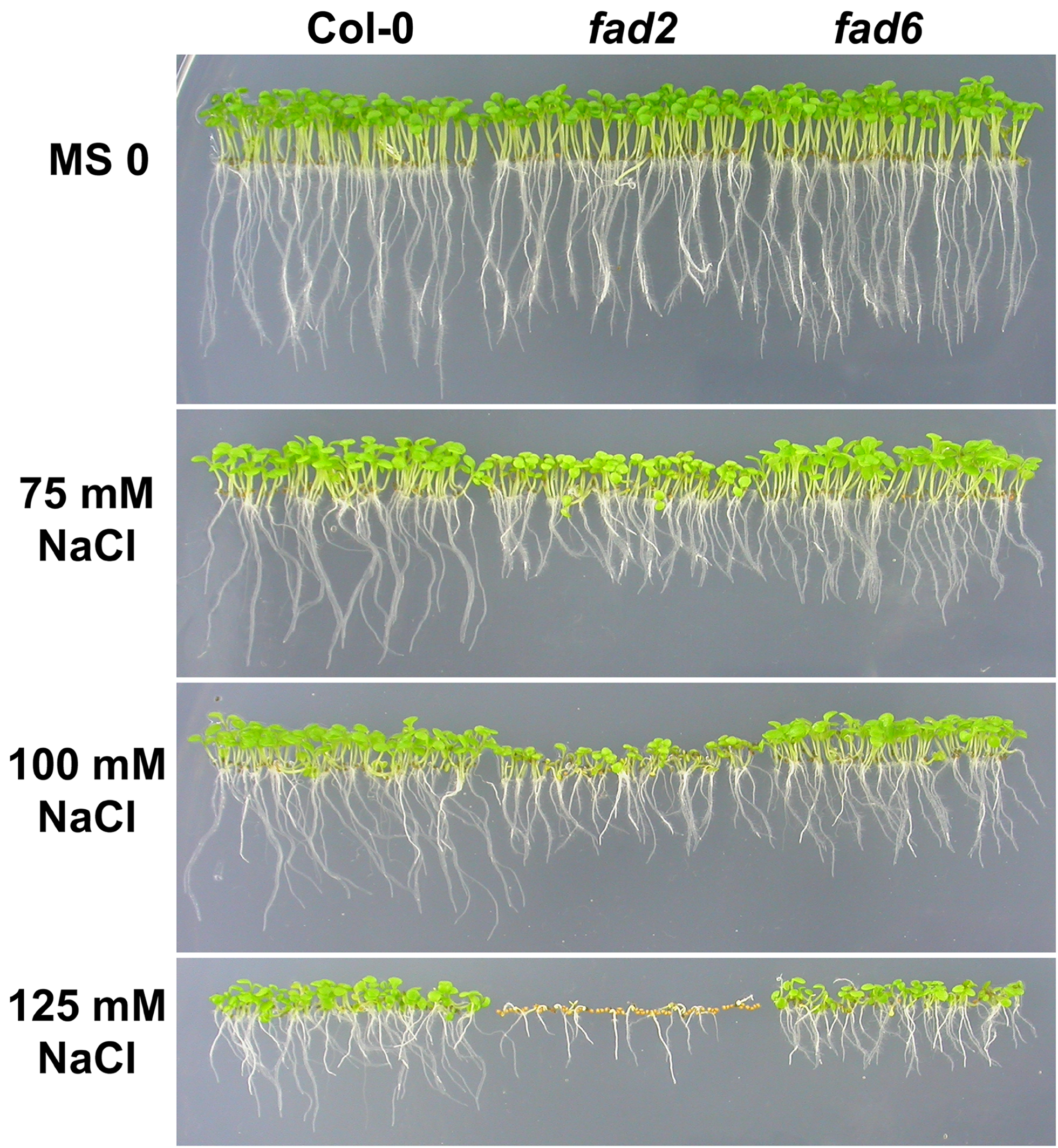

Supplement: Figure S3 — Stress response of Col-0, fad2 and fad6 Mutants. Seeds were sown on MS medium supplemented with different concentrations of NaCl. Photos were taken seven days after stratification, and are representatives of three independent experiments. (TIF) [file pone.0030355.s003.tif]

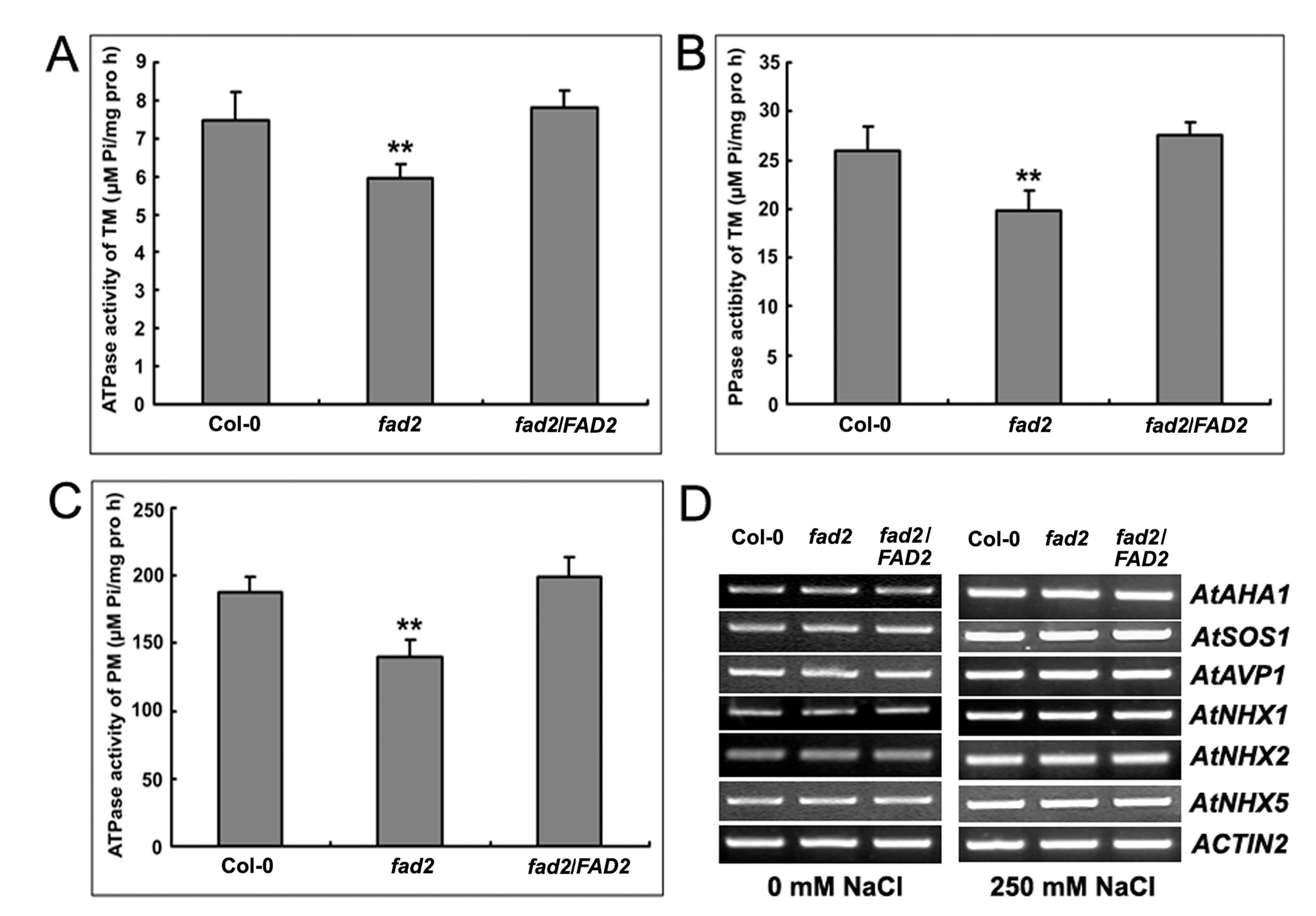

Supplement: Figure S4 — V-ATPase, V-PPase, PM-ATPase Activity and RT-PCR analyses of AtAHA1, AtSOS1, AtAVP1, AtNHX1, AtNHX2 and AtNHX5. Young leaves of 4-week-old plants of Col-0, fad2 and fad2/FAD2 (fad2/FAD2-1) treated with or without 250 mM NaCl for 3 days were used. A-C, Tonoplast and plasma membrane fractions were isolated and enzyme activity assays were performed. D, RT-PCR assays. Total RNA was isolated and RT-PCR was performed with gene specific primers (Table S1). Expression of ACTIN2 was employed as an internal control. (TIF) [file pone.0030355.s004.tif]

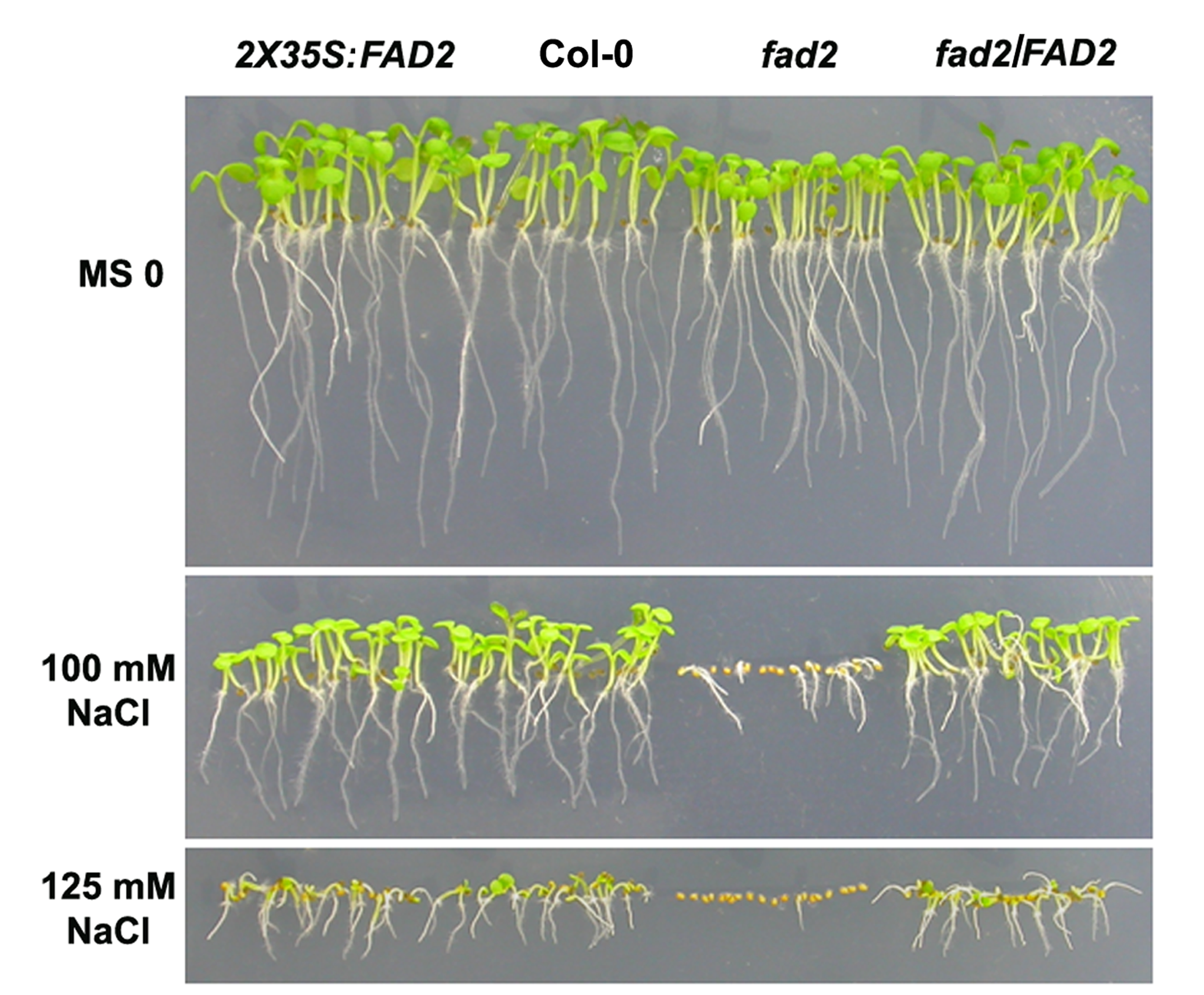

Supplement: Figure S5 — Stress response of p2X35S:FAD2 transgenic plants, Col-0, fad2 and fad2/FAD2 (fad2/FAD2-1). Phenotypes on MS medium supplemented with 100 or 125 mM NaCl. Photos were taken 7 days after the initiation of the treatments, and are representatives of three independent experiments. (TIF) [file pone.0030355.s005.tif]

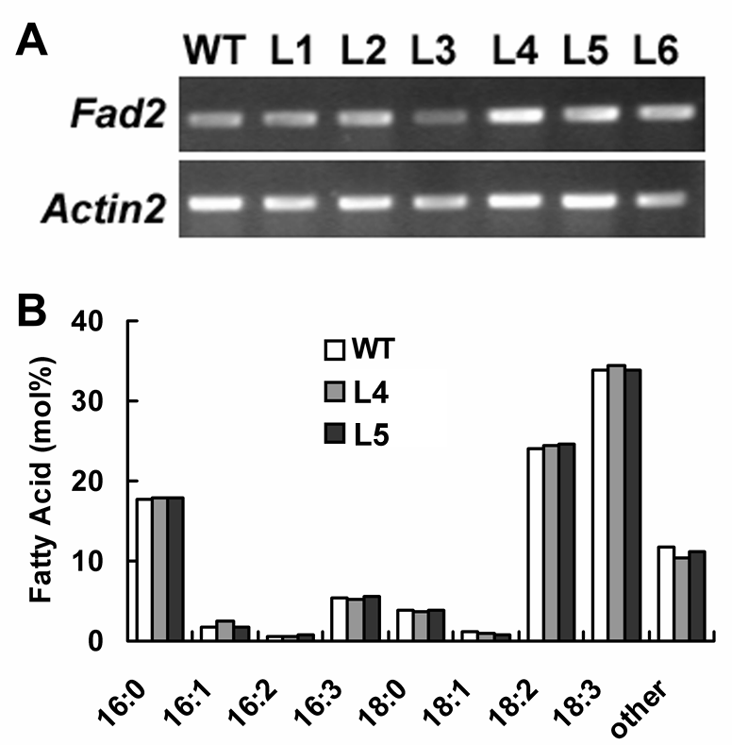

Supplement: Figure S6 — FAD2 expression and fatty acid analyses in wild-type and p2X35S:FAD2 transgenic plants. A, RT-PCR analyses of FAD2 transcripts. B, Fatty acid analyses. WT, wild-type Col-0; L1-L6, different p2X35S:FAD2 transgenic lines. (TIF) [file pone.0030355.s006.tif]
