# Supplementary material for: Arabidopsis Fatty Acid Desaturase FAD2 Is Required for Salt Tolerance during Seed Germination and Early Seedling Growth
Source: PLoS One. 2012 Jan 18;7(1):e30355. doi: 10.1371/journal.pone.0030355 (PMC3261201; doi:10.1371/journal.pone.0030355)
Supplement: Table S1 — Gene specific primer used in this study. (DOC) [file pone.0030355.s007.doc]

Table S1 Gene specific primer used in this study

| *Gene name* | **Sequence of primer** |
| --- | --- |
| *AtAHA1-F* | 5' GCACAACTGGTTGCAACTTTGATC 3' |
| *AtAHA1-R* | 5' GTGATGTCCTGCTGTATCAATGTCC 3' |
| *AtSOS1-F* | 5' CGAAAAAAGAACCAATGAAACTGC 3' |
| *AtSOS1-R* | 5' ATGCGGCGGGAGATGATATAAG 3' |
| *AtAVP1-F* | 5' CCGCAAAACCAGACTACGCC 3' |
| *AtAVP1-R* | 5' GGATACCACCGTGAGTGGCG 3' |
| *AtNHX1-F* | 5' GGTGCCATATTTGCTGCAACAG 3' |
| *AtNHX1-R* | 5' GGATACCGCTCAAGTCGAAAAG 3' |
| *AtNHX2-F* | 5' TCTGGTGGGCTGGTCTAATGAG 3' |
| *AtNHX2-R* | 5' CAGGGACAAAGGGAACGAATC 3' |
| *AtNHX5-F* | 5' GGATGTGGGCACCGATGTTAAC 3' |
| *AtNHX5-R* | 5' ACAATGCCGGAGAGACCAACAC 3' |
| *Actin2-F* | 5' GGAAGGATCTGTACGGTAAC 3' |
| ***Actin2-R*** | 5' GGACCTGCCTCATCATA CT 3' |
